# Efficacy of a Low-Dose Diosmin Therapy on Improving Symptoms and Quality of Life in Patients with Chronic Venous Disease: Randomized, Double-Blind, Placebo-Controlled Trial

**DOI:** 10.3390/nu13030999

**Published:** 2021-03-19

**Authors:** Raffaele Serra, Nicola Ielapi, Andrea Bitonti, Stefano Candido, Salvatore Fregola, Alessandro Gallo, Antonio Loria, Lucia Muraca, Luca Raimondo, Luminița Velcean, Simone Guadagna, Luca Gallelli

**Affiliations:** 1 Interuniversity Center of Phlebolymphology (CIFL), International Research and Educational Program in Clinical and Experimental Biotechnology, University Magna Graecia of Catanzaro, Viale Europa, 88100 Catanzaro, Italy; nicola.ielapi@uniroma1.it (N.I.); turi.fregola@gmail.com (S.F.); ales.gallo@gmail.com (A.G.); 2 Department of Medical and Surgical Sciences, University of Catanzaro, 88100 Catanzaro, Italy; 3 Department of Public Health and Infectious Disease “Sapienza”, University of Rome, 00153 Rome, Italy; 4 Private Office, 80100 Rome, Italy; andrea.bitonti@uniroma1.it; 5 Intensive Care Unit, Pugliese Ciaccio Hospital of Catanzaro, 88100 Catanzaro, Italy; candiste@libero.it; 6 EthosLab SRL, 80100 Catanzaro, Italy; 7 Thoracic Surgery Unit, Annunziata Hospital of Cosenza, 87100 Cosenza, Italy; tonino.loria71@gmail.com; 8 Department of General Medicine, Health Agency of Catanzaro, 88100 Catanzaro, Italy; luciamuraca@libero.it; 9 Department Emergency, Pugliese Ciaccio Hospital of Catanzaro, 88100 Catanzaro, Italy; luca.rai@hotmail.it; 10 Fizio Center, 27, Ulpia Traiana St, 300211 Timișoara, Romania; lumyciungu@yahoo.com; 11 Opera CRO, a Tigermed Company 10 Cozia St., 300209 Timisoara, Romania; simone.guadagna@tigermedgrp.com; 12 Department of Health Sciences, University of Catanzaro, 88100 Catanzaro, Italy; gallelli@unicz.it

**Keywords:** chronic venous disease, symptoms, diosmin, μsmin^®^ Plus, quality of life

## Abstract

Chronic Venous Disease (CVD) is a common medical condition affecting up to 80% of the general population. Clinical manifestations can range from mild to more severe signs and symptoms that contribute to the impairment of the quality of life (QoL) of affected patients. Among treatment options, venoactive drugs such as diosmin are widely used in the symptomatic treatment in all clinical stages. The aim of this study is to determine the effectiveness of a new formulated diosmin in relieving symptoms and improving QoL in patients suffering from CVD. In this randomized, double-blind, placebo-controlled, multicenter clinical study, CVD patients with a Clinical-Etiology-Anatomy-Pathophysiology (CEAP) classification system between C2 and C4 were randomized to receive a bioavailable diosmin (as μsmin^®^ Plus) 450 mg tablet once daily or a placebo for 8 weeks. Clinical symptoms and QoL were monitored using the measurement of leg circumference, visual analogue scale (VAS) for pain, Global Index Score (GIS) and Venous Clinical Severity Score (VCSS). A total of 72 subjects completed the study. From week 4, leg edema was significantly decreased in the active group (*p* < 0.001). An improvement in the VAS score was observed in the active group compared to placebo at the end of treatment (*p* < 0.05). GIS and VCSS scores were significantly improved in the active group at week 8 (*p* < 0.001). No treatment related-side effects were recorded. The results of this study showed that the administration of low-dose μsmin^®^ Plus was safe and effective in relieving symptoms and improving QoL in subjects with CVD.

## 1. Introduction

Chronic Venous Disease (CVD) is a very common clinical condition affecting the Western world. The general prevalence ranges from 10% for adults aged below 30 years up to nearly 80% for subjects aged >70 years old [1], with women appearing to be more affected than men. Moreover, the global prevalence of CVD is expected to escalate dramatically as people are living longer, often with multiple comorbidities [2].

Several genetic and molecular alterations have been found responsible for the onset of CVD, including morphological and functional abnormalities and progression from mild clinical signs, such as reticular and trunk varicose veins, to more advanced and severe signs including leg edema, skin changes and venous ulcers of lower limbs that commonly define the chronic venous insufficiency (CVI) stages [3,4,5,6,7]. Even though CVD is generally not fatal, in severe cases, varicose vein rupture can lead to fatal bleeding [8].

At different stages of this disorder, general practitioners and specialty doctors have to deal with a wide range of symptoms such as leg pain, swelling, itchiness, restless leg syndrome, a burning sensation, and heaviness. These symptoms may be persistent and have a considerable impact on the quality of life (QoL) of many individuals [1,9,10]. Thus, QoL is an important element in the general assessment of any patient.

In the clinical practice, treatment options are based on the individual’s severity of CVD and include non-invasive treatment such as lifestyle changes (e.g., avoiding sitting or standing for long periods, increasing activity level, maintaining a healthy weight, reducing sodium intake), drug administration, surgical and endovascular procedures as well as compression therapies (bandages and elastic stockings) [1,11,12,13].

The focus of available pharmacological treatments is on preventing or resolving the most serious complications such as leg ulceration [14,15,16,17,18], but it is now clear that even in the early state of the disease, mild symptoms may cause daily discomfort to the affected patients [9]. A recent review [19] showed that diosmin, a venoactive drug (VAD), has beneficial effects on CVD symptoms and quality of life (QoL). Diosmin, a naturally occurring flavonoid, is considered a vascular protectant agent due to its anti-inflammatory and antioxidant properties related to the inhibition of several chemical mediators of inflammation (cytokines, metalloproteases, histamine, serotonin, etc.) and due to its specific phlebotonic action on the venous wall [14].

Recently, a newly formulated diosmin ingredient, μsmin^®^ Plus (Giellepi S.p.A., Lissone, Italy), was developed to overcome diosmin’s poor oral bioavailability. This product exhibited a 4-fold increase in relative bioavailability compared to micronized diosmin in rats [20], and resulted in 9.4-fold higher relative bioavailability compared to micronized diosmin following oral ingestion in healthy subjects [21].

In light of these promising results, this study was designed to test the efficacy and safety of µsmin^®^ Plus in a setting of standard clinical practice where the administration of VADs in patients suffering from CVD is often integrated with lifestyle counselling.

## 2. Methods

### 2.1. Study Design

This randomized, double-blind, placebo-controlled, multicenter, 8-week clinical trial was aimed at the evaluation of the efficacy and safety of a commercially available dietary supplement (µsmin^®^ Plus, a diosmin preparation with enhanced bioavailability) in alleviating symptoms of CVD. The main objectives of the study were the changes in calf circumference and the impact of the active treatment on quality of life, while secondary objectives were the evaluation of CVD-related symptoms including pain, investigator/patient treatment satisfaction and safety. During the study, a total of 3 visits were scheduled. Screening and baseline assessment visit (visit 1; day −14 to day 0): vital signs and general health condition were checked through physical examination; medical history, concomitant medications and baseline characteristics of each patient (including calf circumference, visual analogue scale (VAS) score, Venous Clinical Severity Score (VCSS) and quality of life) were recorded; eligible patients were asked to sign the informed consent. 

Intermediate follow-up visit (visit 2; week 4 ± 4 days from recruitment): subject evaluation and symptoms assessment were performed. Adverse effects, if any, were recorded. 

End of the study visit (visit 3; week 8 ± 4 days from recruitment): final subject evaluation and symptoms assessment were performed. Adverse effects, if any, were recorded. 

The product administration protocol consisted of one coated tablet, once daily, of µsmin^®^ Plus (Giellepi S.p.A., Lissone, Italy) equivalent to 450 mg diosmin. µsmin Plus^®^ is a well-established oral flavonoid with venoprotective properties consisting of 80% micronized diosmin and 90% total flavonoids.

This study was conducted according to the guidelines included in the Declaration of Helsinki and all the procedures were approved by the Institutional Review Board SCM Gados, Timisoara, Timis, Romania (Approval Protocol OPGIE/0119/FS). Written informed consent was obtained from all subjects before inclusion in the study. The trial was registered in the clinicaltrials.gov database (NCT04101201). 

### 2.2. Patients

Eligible patients were male and female aged ≥18 and ≤60 suffering from CVD with CVI grade C2–C4 on the Clinical-Etiology-Anatomy-Pathophysiology (CEAP) classification system [22]. Exclusion criteria were patients with vascular diseases other than CVD, diabetes, or blood disorders; lower limb edema (cardiac, renal, or hepatic origin); lower limb arterial disease; and also metabolic, neurological, or orthopedic problems, including traumas and prior amputation, arthritis, neuropathy, that may mimic venous symptoms, or other conditions such as recent childbirth, recent vein surgery, or deep or superficial venous thrombosis of the lower limbs during the previous 6 months; obese subjects (BMI > 30); hypersensitivity to active principles contained in the tested dietary supplement (diosmin); smoker status (≥10 cigarettes/day); concomitant or previous history of addiction to alcohol, excessive use of spices, or drug abuse; pregnant women, nursing mothers, or women (only if childbearing potential) not using adequate methods of contraception; participation in an interventional clinical study in the previous 30 days; and the presence of any clinically significant medical condition in order to preclude the patient’s inclusion in the study. 

Patients fulfilling the eligibility criteria were enrolled and randomized by a computer method with equal distribution (allocation ratio 1:1) to receive either diosmin 450 mg tablets as µsmin^®^ Plus or matched placebo tablets. According to the summary of product characteristics provided by the manufacturer, one tablet once daily was administered to each subject for 8 consecutive weeks. 

Phlebotonics and other CVD-related treatments were not allowed during the study. There was no restriction on treatments taken previously by subjects for medical conditions not related to this study protocol.

### 2.3. Primary Endpoints

Primary outcomes investigated were change in mid-calf circumference (measured by investigator in cm) of each affected leg and quality of life (QoL) assessed by Global Index Score (GIS) calculated through the CIVQ-20 questionnaire [23]. The Chronic Venous Insufficiency quality of life Questionnaire (CIVIQ-20) is an instrument with 20 questions each with 5 possible answers (1 to 5), the minimum possible score being 20 and the maximum 100. GIS was calculated based on CIVIQ-20 questionnaire score following the formula: GIS = 100 − ([Final CIVIQ-20 score − 20]/80) × 100.

### 2.4. Secondary Endpoints 

The following outcomes were recorded:

- VAS scale for pain [24]. Using a ruler, the score is determined by measuring the distance (mm) on the 100 mm line between the “no pain” anchor and the patient’s mark, providing a range of scores from 0 to 100. A higher score indicates greater pain intensity. The following cut points on the pain VAS have been recommended: no pain (0–4 mm), mild pain (5–44 mm), moderate pain (45–74 mm), and severe pain (75–100 mm). At each visit, patients were asked to mark a vertical sign on the scale to assess the intensity of pain. For pain assessment, the investigator measured with a ruler the distance between point 0 and the marked line by the patient.
- The Venous Clinical Severity Score (VCSS) [25] includes 9 attributes of venous disease, each scored on a severity scale from 0 to 3. (Absent = 0, Mild = 1, Moderate = 2, Severe = 3). In order to generate a dynamic score, VCSS attributes are scored individually. These attributes include skin changes and pigmentation, inflammation and induration, and ulcers (including number, size, and duration), compression therapy, and pain. Total score (ranging between 0 and 27) was calculated by summing scores from all attributes. A decreasing score means a relief of symptoms.
- Investigator Global Assessment of Efficacy—Investigators made the global assessment regarding the treatment efficacy using a 4-point scale (1 = excellent, 2 = good, 3 = fair, 4 = poor), at visit 2 and 3.
- Patient Global Assessment of Efficacy—Patients made the global assessment regarding the treatment efficacy using a 5-point scale (1 = very satisfied, 2 = satisfied, 3 = adequate, 4 = unsatisfied, 5 = very unsatisfied), at visit 2 and 3.
- Continuation with investigational product—The evaluation of subject options about continuing with the investigational product after the study termination was assessed at week 8 (final visit) for both groups. This variable is treated as a categorical variable and described by percentages and frequencies for each allocation group.
- Evaluation of symptoms relief—Evaluation of symptoms relief was assessed by evaluating the percentage of subjects who experienced symptoms relief within the first week of intake, within 2 weeks of intake, or more than 2 weeks of intake.
- Investigator Global Assessment of Safety—Investigators made the global assessment regarding the safety of treatments using a 4-point scale (1 = very good safety, 2 = good safety, 3 = moderate safety and 4 = poor safety), at visit 2 and 3.

### 2.5. Statistical Analysis

Quantitative variables (i.e., demographic) if normally distributed were described through mean ± Standard Deviation (SD), otherwise median, minimum, maximum, and interquartile ranges were shown. Qualitative variables were evaluated using frequencies and percentages. To evaluate changes over time before and after the administration, Paired *t*-tests (if applicable) or Wilcoxon’s signed-rank sum tests were applied for quantitative variables, while McNemar’s tests were used to evaluate changes for binary variables and symmetry tests were performed to evaluate changes for qualitative (not binary) variables. The quality and completeness of the collected data was evaluated preliminarily compared to data analysis. If a subject was missing information for one or more variables, even after the resolution of its query, the missing data were not replaced. If a subject had been involved in the violation of inclusion/exclusion criteria, the respective data were excluded from the analysis. 

## 3. Results

### 3.1. Patients

Out of 73 subjects screened, 72 were considered eligible for the completion of this study. Subjects were randomly assigned either to the µsmin^®^ Plus group (*n* = 37) or the placebo group (*n* = 35). All 72 subjects enrolled completed the study and all of them were included in the intention to treat (ITT) and safety population analysis. Subject demographics are presented in Table 1 and revealed no significant differences (*p* > 0.05) in baseline demographic and anthropometric measures. It is noteworthy that one subject from the μsmin^®^ Plus group and one subject from the placebo group could not be investigated at visit 2 and 3 due to travel restrictions caused by the COVID-19 pandemic. Therefore, 36 subjects in the μsmin^®^ Plus group and 34 subjects in the placebo group were investigated at visit 2 and 3.

### 3.2. Efficacy of Study Treatments

#### Primary Efficacy Outcomes: Circumference of Each Affected Leg (Calf) and Quality of Life Assessed by Global Index Score

**Figure 1:**
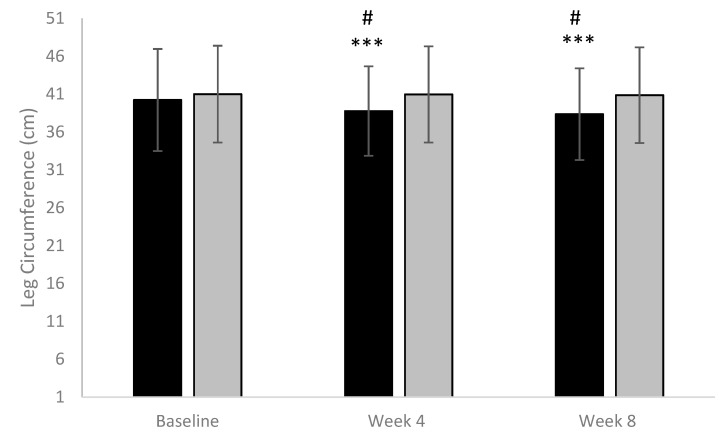
Leg Circumference. (Black column: active; Grey column: placebo). *** *p* < 0.001 vs. baseline; # *p* < 0.05 active vs. placebo.

When compared to the placebo group, the active group showed a statistically significant reduction in leg circumference either after 4 (*p* = 0.033) or 8 weeks (*p* = 0.016) (Figure 1); in particular, the average decrease in % was: 3.63% and 4.67% after 4 weeks and after 8 weeks in the active group; while it was 0.07% and 0.32% after 4 weeks and after 8 weeks in the placebo group.

**Figure 2:**
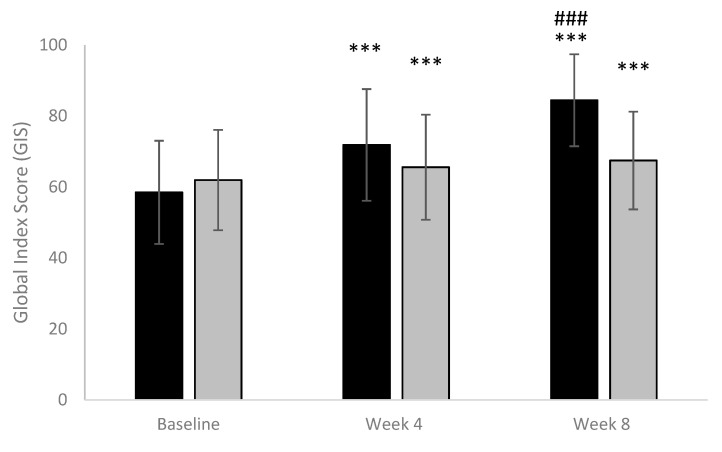
Global Index Score. (Black column: active; Grey column: placebo). *** *p* < 0.001 vs. baseline; ### *p* < 0.001 active vs. placebo.

In both groups of patients, GIS improved from baseline to the end of treatment from 58.48 (±14.55) to 84.43 (±12.95) and from 61.96 (±14.13) to 67.46 (±13.77) in the active group and placebo group, respectively. At the end of treatment (week 8), a significant increase in GIS (*p* < 0.001) was found in the active group in comparison to the placebo group. Results are shown in Figure 2.

### 3.3. Secondary Efficacy Outcomes

**Figure 3:**
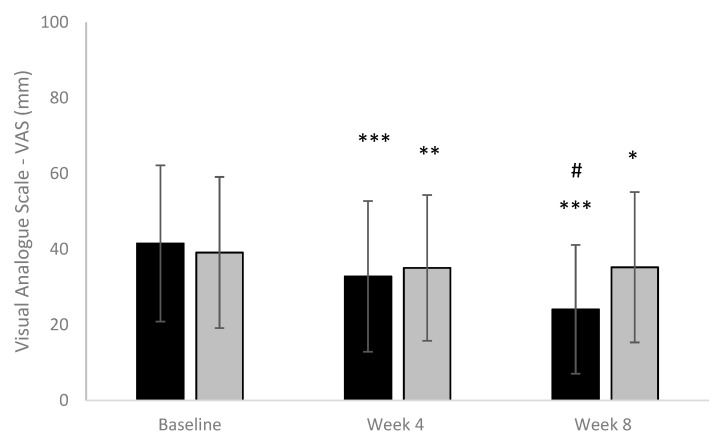
Visual Analogue Scale (VAS) for pain. (Black column: active; Grey column: placebo). * *p* < 0.05 vs. baseline; ** *p* < 0.01 vs. baseline; *** *p* < 0.001 vs. baseline; # *p* < 0.05 active vs. baseline.

VAS scores decreased progressively from baseline to end of the study both in the active and placebo group. At the end of treatment, a reduction in VAS score was found statistically significant (*p* = 0.014) in the µsmin^®^ Plus group compared to the placebo group. Results are shown in Figure 3.

**Figure 4:**
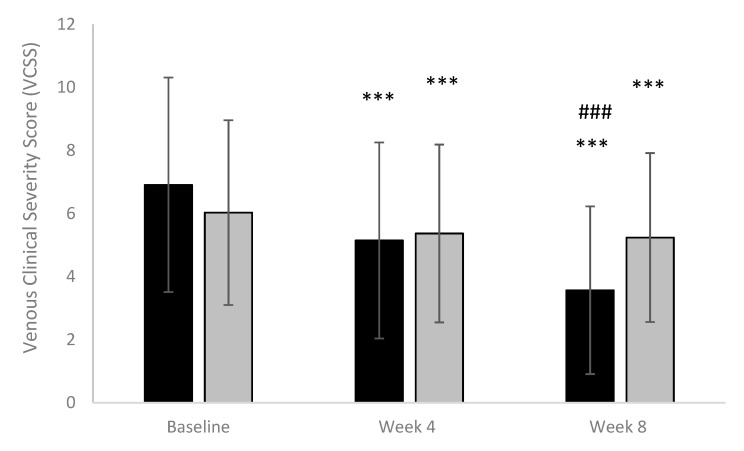
Venous Clinical Severity Score. (Black column: active; Grey column: placebo) *** *p* < 0.001 vs. baseline; ### *p* < 0.001 active vs. baseline.

Both µsmin^®^ Plus and placebo treatment reduced the symptoms’ severity significantly after 4 weeks and 8 weeks compared to baseline (*p* < 0.001). At the end of treatment, µsmin^®^ Plus showed a significant improvement in Venous Clinical Severity Score (VCSS) compared to the placebo (*p* < 0.001). Results are shown in Figure 4.

The Investigator Global Assessment of Efficacy (IGAE) between groups is summarized in Table 2. The efficacy of treatment with µsmin^®^ Plus was already evaluated as excellent or good by the investigators compared to the placebo after 4 weeks.

The evaluation of Patient Global Assessment of Efficacy (PGAE) is reported in Table 3. At the end of the study, all patients were satisfied or very satisfied with the treatment with µsmin^®^ Plus. 

The evaluation of symptoms relief was assessed by evaluating the following symptoms: pain, varicose veins, edema, inflammation, skin pigmentation, induration, number of active ulcers, active ulcers duration, active ulcer diameter. Symptoms relief is summarized in Table 4. The results show that 68% of patients who ingested the investigational product experienced a reduction in symptoms within 2 weeks of intake vs. only 11% of patients in the placebo group.

### 3.4. Safety Outcomes

No adverse events or serious adverse events have been reported during the clinical trial.

The Investigator Global Assessment of Safety (IGAS), which is summarized in Table 5, was reported “very good” in more than 97% of patients who ingested the investigated dietary supplement.

## 4. Discussion

Chronic Venous Disease (CVD) determines morphological and functional abnormalities of the leg venous system, causing a broad range of long duration signs and symptoms. Understanding the complex pathophysiology of CVD is pivotal to support the identification of novel therapeutic targets in order to effectively relieve CVD clinical manifestations, thus improving the QoL of affected patients. Due to their potential in inhibiting inflammation pathways and their ability to interact with key molecular targets, VADs have shown consistent efficacy across all CVD stages [26,27,28,29,30,31,32]. 

CVD related symptoms and signs such as pain, cramps, leg heaviness, paresthesia, itching, and edema were relieved to some extent by treatments with VADs [1,26]. 

Diosmin, a flavonoid commonly found in citrus fruits, has been widely used and prescribed for more than 30 years as a VAD in CVD for its phlebotonic effect known to improve venous tone, stabilize capillary permeability and increase lymphatic drainage, and also for its well-established safety profile [1]. It has been recently shown that a possible mechanism of action involved in the improvement of CVD after oral diosmin treatment may be a significant decrease in proinflammatory mediators such as IL-6 and TNF-α and pro-angiogenetic factors [33]. 

Flavonoids, including diosmin, are poorly absorbed following oral administration. They are usually hydrolyzed in small phenolic compounds by the enzymes from the intestinal bacteria [34]; these small molecules can cross the intestinal wall easily and reach higher plasma levels than parent flavonoids, and hence can be responsible for the biological effect of parent flavonoids such as antiplatelet effect [35,36,37]. After liver metabolism, diosmetin, which is the aglycone of diosmin, is rapidly excreted in the bile as glucuronide and sulphate conjugates [38].

For overcoming diosmin’s poor bioavailability following oral ingestion, several attempts have been made, of which the reduction in particle size (i.e., micronization) is the most commonly used, to increase flavonoids’ absorption and bioavailability. Although the plasma levels are generally improved after the oral administration of micronized diosmin, concentrations are often low and highly different across published studies [35,39,40]. A micronized flavonoid fraction (MPFF), consisting of 90% diosmin and 10% hesperidin, has been successfully used in Europe as a drug or nonprescription dietary supplement for the treatment of chronic venous insufficiency with a treatment regimen of 1000 mg/day given in two divided doses [19,41,42,43].

This clinical investigation was designed to investigate the effectiveness of a low-dose diosmin treatment consisting of 450 mg of diosmin delivered once a day as μsmin^®^ Plus for alleviating typical symptoms associated with CVD. Subjects were randomized to receive either the treatment formulation (1 tablet/day) or a placebo (1 tablet/day) for 56 consecutive days (8 weeks). Different measurements, scales, and questionnaires were used to quantify the potential beneficial effects of μsmin^®^ Plus on the symptomatology of CVD. Overall, the results clearly indicate that the investigational product is effective in decreasing leg circumference, pain (on moving and standby), and a wide array of symptoms commonly associated with CVD. Nevertheless, following 8 weeks of daily administration, the active product was not associated with any AE/SAEs occurrence, proving its excellent safety. In general, diosmin is considered a safe substance. Mild side effects such as gastralgia, diarrhea, and abdominal pain were only reported in a few cases [43]. We speculate that the absence of any side effects is related to the low-dosage regimen used in our study (i.e., 570 mg µsmin^®^ Plus equal to 450 mg diosmin). In fact, typical diosmin-related side effects are usually seen with treatment between 1000 and 3000 mg/day of MPFF.

A previous study [33] performed with a population similar to the one of our investigation, consisting of 35 CVD patients (C2–C4), showed that treatment with 600 mg of diosmin twice daily for 3 months produced similar results, reducing both edema assessed by leg circumference and pain assessed by VAS, without any side effects. Such evidence agrees with the results of our study both in terms of clinical efficacy and safety. 

Current clinical evidence indicates that MPFF at the dose of 500 mg twice per day or 1000 mg once per day may be sufficient for improving symptoms in CVD patients. Interestingly, μsmin^®^ Plus has been proven effective at a low-dose regimen (450 mg once per day), thus confirming the data obtained from previous pharmacokinetic studies [19,21]. Moreover, as a confirmation of the better bioavailability of the tested formulation, 68% of patients in the active group experienced a significant symptom reduction already after 2 weeks of intake. 

From a practical point of view, a single daily dose may improve patient adherence to the medical treatment as already shown in other treatment protocols [44]. This becomes even more relevant among patients on long-term treatments.

It is important to note that the content of hesperidin in μsmin^®^ Plus is negligible (i.e., 1% of total diosmin content) and significantly lower compared to other studied compounds such as micronized purified flavonoid fraction (MPFF) [19]. Therefore, it can be speculated that hesperidin does not play a key role when co-administered with diosmin.

Chronic Venous Disease has been associated with the significant deterioration of general and health-related QoL. In fact, pain, swelling, leg discomfort and severe skin changes may lead to a progressive impairment of QoL not only associated with physical items but also with emotional/mental health items. As a result, the improvement of QoL can be viewed as the main therapeutic target in CVD [45]. Although not evaluated in this study, an improvement in sleep quality is also conceivable as some of the symptoms of chronic venous insufficiency occurring at night, such as heaviness and leg cramps [46], were markedly improved by μsmin^®^ Plus. 

CVD’s treatment options have evolved in recent years. However, clinicians need to have in their arsenal additional therapeutic products for improving the everyday life of their patients. In this context, several tools can be used to evaluate the safety and effectiveness of new treatments on QoL [47]. In this study, QoL assessed by GIS and calculated through the CIVIQ-20 questionnaire was significantly improved at the end of treatment in the active group compared to the placebo group. Such results agree with those published previously, obtained after treatment with micronized diosmin or MPFF at higher dosages (1000 mg/day) or longer periods (up to 6 months or longer) [19,48,49].

One limitation of the current study was that venous calcifications were not measured, and therefore it is not possible to evaluate this issue within the outcomes of our study. 

## 5. Conclusions

Based on findings from the current study, we concluded that μsmin^®^ Plus, a diosmin preparation with enhanced bioavailability, is safe and effective for improving CVD symptomatology after 8 weeks of treatment in subjects with CEAP clinical classification C2–C4.

## Figures and Tables

**Table 1:** Demographics.

Variables	Treatment	*n*	Mean (SD)	Median	1st Quartile	3rd Quartile
**Age (years)**	µsmin^®^ Plus	37	53.32 (5.29)	54	32	60
	Placebo	35	52.63 (7.38)	55	30	60
**Weight (kg)**	µsmin^®^ Plus	37	73.54 (10.42)	72	50	97
	Placebo	35	69.34 (7.91)	69	50	81
**Height (cm)**	µsmin^®^ Plus	37	170.35 (8.35)	170	156	190
	Placebo	35	167.54 (6.46)	167	155	183
**BMI (kg/m^2^)**	µsmin^®^ Plus	37	25.24 (2.16)	25.06	20.2	29.30
	Placebo	35	24.70 (2.50)	24.45	19.03	29.38
**Heart rate (bpm)**	µsmin^®^ Plus	37	76.08 (7.29)	75	60	90
	Placebo	35	76.03 (7.25)	76	60	90
**Sex**	µsmin^®^ Plus	Males: 10 Females: 27	-	-	-	-
	Placebo	Males: 5 Females: 30	-	-	-	-

Legend: SD: standard deviation; BMI: body mass index; bpm: beats per minute.

**Table 2:** Investigator Global Assessment of the Efficacy (IGAE) after 4 weeks and 8 weeks of treatment.

Week	Treatment	*n* (%)	Excellent	Good	Fair	Poor
**Week 4**	µsmin^®^ Plus	37 (100)	6 (16.22)	28 (75.68)	3 (8.11)	0
	Placebo	35 (100)	1 (2.86)	3 (8.57)	8 (22.86)	23 (65.71)
**Week 8**	µsmin^®^ Plus	37 (100)	19 (51.35)	17 (45.95)	1 (2.7)	0
	Placebo	35 (100)	0	3 (8.57)	3 (8.57)	29 (82.86)

**Table 3:** Patient Global Assessment of the Efficacy (PGAE) after 4 weeks and 8 weeks of treatment.

Week	Treatment	*n* (%)	Very Satisfied	Satisfied	Adequate	Unsatisfied	Very Unsatisfied
**Week 4**	µsmin^®^ Plus	37 (100)	4 (10.81)	22 (59.46)	11 (29.73)	0	0
	Placebo	35 (100)	1 (2.86)	2 (5.71)	9 (25.71)	21 (60.00)	2 (5.71)
**Week 8**	µsmin^®^ Plus	37 (100)	20 (54.05)	17 (45.95)	0	0	0
	Placebo	35 (100)	0	5 (14.29)	2 (5.71)	23 (65.71)	5 (14.29)

**Table 4:** Evaluation of symptoms relief.

Treatment	*n* (%)	Within the First Week of Intake	Within 2 Weeks of Intake	After More than 2 Weeks of Intake	No Improvement
µsmin^®^ Plus	37 (100)	4 (10.81)	25 (67.57)	7 (18.92)	1 (2.70)
Placebo	35 (100)	0	4 (11.43)	6 (17.14)	25 (71.43)

**Table 5:** Investigator Global Assessment of Safety (IGAS) after 4 weeks and 8 weeks of treatment.

Week	Treatment	*n* (%)	Very Good	Good	Moderate	Poor
**Week 4**	µsmin^®^ Plus	37 (100)	36 (97.30)	1 (2.70)	0	0
	Placebo	35 (100)	24 (68.57)	11 (31.43)	0	0
**Week 8**	µsmin^®^ Plus	37 (100)	36 (97.30)	1 (2.70)	0	0
	Placebo	35 (100)	31 (88.57)	4 (11.43)	0	0

## Data Availability

The data that support the findings of this study are available in the Trial Master File at the sponsor’s archive.

## Abbreviations

ANOVA	Analysis of variance
AE	Adverse Event
CEAP	Clinical-Etiology-Anatomy-Pathophysiology
IC	Informed Consent
IGAE	Investigator Global Assessment of Efficacy
IGAS	Investigator Global Assessment of Safety
IP	Investigational Product
ITT	Intention to treat
PGAE	Patient Global Assessment of Efficacy
QoL	Quality of Life
SAE	Serious Adverse Event
SD	Standard Deviation
VAS	Visual Analogue Scale
